# Exploring the Impacts of Anthropogenic Disturbance on Seawater and Sediment Microbial Communities in Korean Coastal Waters Using Metagenomics Analysis

**DOI:** 10.3390/ijerph14020130

**Published:** 2017-01-27

**Authors:** Nam-Il Won, Ki-Hwan Kim, Ji Hyoun Kang, Sang Rul Park, Hyuk Je Lee

**Affiliations:** 1 K-Water Institute, Korea Water Resources Corporation, Daejeon 34350, Korea; niwon12130208@gmail.com; 2 Gencube, Seoul 10110, Korea; seqkim@naver.com; 3 Korean Entomological Institute, Korea University, Seoul 02841, Korea; jihyounkang@korea.ac.kr; 4 Estuarine and Coastal Ecology Laboratory, Department of Marine Life Sciences, Jeju National University, Jeju 63243, Korea; srpark@jejunu.ac.kr; 5 Molecular Ecology and Evolution Laboratory, Department of Biological Science, Sangji University, Wonju 26339, Korea

**Keywords:** coastal ecosystem, environmental DNA, metagenomics, microbial community, operational taxonomic unit (OTU), sand mining

## Abstract

The coastal ecosystems are considered as one of the most dynamic and vulnerable environments under various anthropogenic developments and the effects of climate change. Variations in the composition and diversity of microbial communities may be a good indicator for determining whether the marine ecosystems are affected by complex forcing stressors. DNA sequence-based metagenomics has recently emerged as a promising tool for analyzing the structure and diversity of microbial communities based on environmental DNA (eDNA). However, few studies have so far been performed using this approach to assess the impacts of human activities on the microbial communities in marine systems. In this study, using metagenomic DNA sequencing (16S ribosomal RNA gene), we analyzed and compared seawater and sediment communities between sand mining and control (natural) sites in southern coastal waters of Korea to assess whether anthropogenic activities have significantly affected the microbial communities. The sand mining sites harbored considerably lower levels of microbial diversities in the surface seawater community during spring compared with control sites. Moreover, the sand mining areas had distinct microbial taxonomic group compositions, particularly during spring season. The microbial groups detected solely in the sediment load/dredging areas (e.g., *Marinobacter, Alcanivorax, Novosphingobium*) are known to be involved in degradation of toxic chemicals such as hydrocarbon, oil, and aromatic compounds, and they also contain potential pathogens. This study highlights the versatility of metagenomics in monitoring and diagnosing the impacts of human disturbance on the environmental health of marine ecosystems from eDNA.

## 1. Introduction

The marine ecosystem is the largest, most diverse, complex, and influential for all life on Earth, including humans. It plays a fundamental role in the functioning of the global ecosystem and services such as providing the balance of global water and geochemical cycles, the buffering of global climate change, and the fisheries production for human society [1,2]. However, the global ocean health has been under severe threat, particularly recently, predominantly due to anthropogenic pressure [3,4]. Various alarming signals such as decreasing fisheries production, oxygen depletion, and acidification have been identified in the global ocean [4]. The coastal ecosystem, in particular, might be the most critical component of ocean health since it is affected by multifaceted stressors including various anthropogenic activities (e.g., coastal developments) as well as natural variabilities. The ecosystem resilience following (human) disturbance is one of the essential components in securing a sustainable ecosystem and it has been evaluated by determining the function, structure, and biodiversity of the target ecosystem [5].

Marine microbial communities are complex and highly diverse; on average, ocean water contains one million microorganisms per milliliter, with thousands of unique microbial taxonomic groups [6]. They play a pivotal role in a number of ecological processes such as biogeochemical or nutrient cycling and energy flow, serving as regulators of oxygen, carbon, and nutrient dynamics (i.e., ecosystem functioning) [7]. In shallow coastal areas, microbial activities in bottom sediments and water column are particularly crucial for understanding coastal ecological processes, e.g., coastal benthic-pelagic coupling [8].

Evaluation of the structure and diversity of microorganisms or bacterial communities in coastal ecosystems has recently been suggested to be critical to gauge their environmental status, particularly for those that have been affected by human-driven stressors [9,10]. Microbial organisms can be a reliable indicator for detecting or diagnosing changes in seawater ecosystems because they are known to be sensitive to hydrologic and water quality changes exhibiting a rapid response to those changes (e.g., [11]). They can also rapidly respond to seasonal fluctuations, including phytoplankton abundance, grazing pressure, and nitrogen, phosphate and silica concentrations [12,13,14]. Moreover, environmental parameters such as temperature, which is dependent on seasonal changes, have an effect on the bacterial community [12,15]. Therefore, patterns of a detectable change in the structure and diversity of microbial communities in the coastal ecosystems could reflect the “in situ” effects of various environmental parameters (reviewed by [16]).

**Figure 1:**
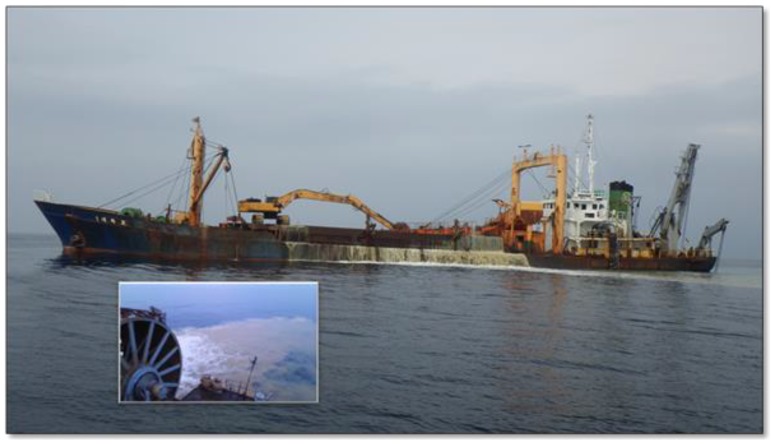
Photo of sediment load and dredging, i.e., sand mining activity in southern coastal waters of Korea. The designated sand mining areas are approximately 80 m deep and 100 km away from the main land of Korea (Figure 2). Overflowing of pumped bottom sand or sediment is remarkable around the ship and the spreading of overflowed sands is indicated as seen in the small photo.

In Korean coastal waters, marine sand mining activities (e.g., bottom sediment load or dredging) are routinely undertaken to supply sand materials for construction purposes, because of the recently increasing coastal developments (Figure 1). These sand mining events continually introduce about 90-m deep bottom sediments into deep, middle, and especially surface water layers, leading to spreading of fine sediments up to approximately 10 km away, depending on the oceanographic conditions (N. Won, unpublished data). Such a physical disturbance of underwater sediments causes turbidity in the seawater, which might have a devastating effect, particularly on phototrophic organisms that require sunlight for primary production [17]. The sand mining activity also entails the impact and risks on the microbial community involved in toxicity, nutrient enrichment, and bioaccumulation by heavy metals and organic pollutants [17]. Heavy metals and organic compounds contamination through sand mining or dredging activities stems from polluted bottom sediments [18,19]. Continuous sand mining events ultimately lead to a decrease in fisheries production [18,20], and therefore its public concerns have been increased in local communities in Korea. However, the ecological impacts on the marine ecosystem around the sand mining areas have not been assessed yet, primarily due to logistic difficulties and remote locations of target areas, more than 100 km away from the main land. To our knowledge, microbial communities have never been evaluated in terms of the possible impacts of sand mining events on the target ecosystem in Korea, although there are a few studies that examined the effects of sediment dredging on the marine bacterial community in different regions of the world (e.g., the North Sea [18,19]).

Metagenomics involves sequencing of the total DNA extracted from an environmental sample (i.e., environmental DNA; eDNA) containing several different organisms [21]. The metagenomics approach has recently been proven to be a powerful tool to gauge “hidden” ecosystem biodiversity, which was not possible in the past, and a number of studies have been conducted using this approach to examine the marine microbial communities [13,14,22,23,24,25,26,27]. Next-generation sequencing (NGS) techniques for phylogenetically informative markers such as the ribosomal RNA (rRNA) genes have enabled assessments of the taxonomic diversity and structure of marine microbial communities [12,13,14]. Furthermore, it has recently been suggested that “marine metagenomics” is a reliable and novel method for assessing and monitoring environmental health status in the marine ecosystem [28]. Nevertheless, relatively few studies have so far been conducted using metagenomic DNA sequencing (e.g., 16S rRNA) to directly assess the impacts of human activities on the structure and biodiversity of microbial communities in the marine ecosystems [9,10,18,19].

In the present study, we aimed to explore the marine natural microbial communities and the impacts of sand mining activities on these environments in Korean waters using NGS-based DNA sequencing of 16S rRNA gene. The specific objective of this study was to assess whether human-driven coastal developments, i.e., sand mining activities, have significantly influenced seawater and sediment microbial ecosystems. For this purpose, we analyzed and compared the structure and diversity of microbial communities between coastal regions with continuous sand mining activities vs. unaffected natural environments (i.e., control) during two different seasons (spring and autumn). This study will help to better understand the mechanisms that shape bacterial diversity in this particular ecosystem.

## 2. Materials and Methods

### 2.1. Sample Collection, Processing, and eDNA Isolation

**Figure 2:**
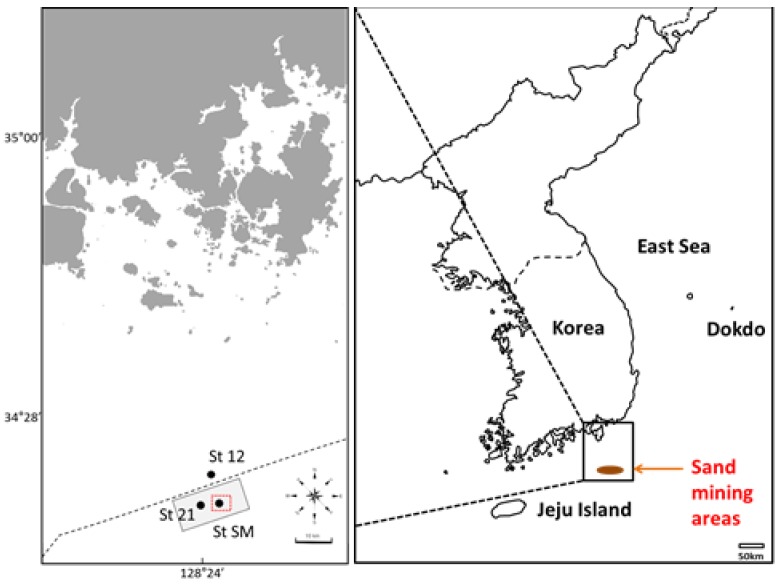
Maps showing the sampling sites of seawaters and sediments from southern coastal waters of Korea. St. 21 (34°11.55′ N; 128°22.55′ E) and St. SM (34°11.17′ N; 128°24.50′ E) represent ‘sand mining’ site, and St. 12 (34°15.05′ N; 128°23.68′ E) ‘control’ site. The red square area on the left map represents the authorized on-going sand mining area (St SM). The grey area on the left map represents the marine sand research area for mining (St. 21). The dashed line indicates the Korean coastal waters up to 12 km from the main land.

Seawater and sediment samples were obtained from two different marine environments (“sand mining” site (site 21, site SM) and “control” site (site 12)) in the open oceans of the South Sea in Korea using the Van Dorn water sampler (KC Denmark, Silkeborg, Denmark) and the Van Veen grab sampler (KC Denmark), respectively (Figure 2; Table 1). We chose site 12 as the control since loaded and suspended fine sediments by sand mining events were observed to spread up to about 10 km away by analyzing in-situ data measured by acoustic Doppler current profiler (ADCP, SonTek, San Diego, CA, USA) during the present study (N. Won, unpublished data). Therefore, site 12 was presumed to represent a “natural” environment. The collected seawater and sediment samples were temporarily stored in sterile acid-washed Nalgene (Rochester, NY, USA) bottles and Falcon (Pittsburgh, PA, USA) tubes, respectively. Nalgene plastic bottles were autoclaved and rinsed with seawater prior to sampling. Sampling was performed during two different periods of April and October 2015.

Water samples (20 L) were collected twice (two technical replicates) at each of three different depths—surface, middle (30 m in depth), and deep (60 m)—for each of the study sites to examine if the structure of microbial communities changed with water depth. Seawater samples were stored at 4 °C for less than 12 h unless being used immediately for filtration. The sediment samples were also collected at identical sites as seawater samples, temporarily stored at 4 °C, and then transported to the laboratory at −80 °C for DNA extraction. Unfortunately, however, we were not able to collect all the samples (e.g., sediment samples were not obtained from the control site during October 2015).

To isolate eDNA from seawater samples, 20 L of seawater from each sample was filtered through a 90-mm diameter, 2.5-µm GF/A filter (Whatman, Sigma-Aldrich, Darmstadt, Germany) using Masterflex I/P tubing pump (Millipore, Darmstadt, Germany) in situ, immediately after collection, to reduce eukaryotic cell abundances and maximize the proportion of prokaryotic cells, as suggested by [23]. Then, the filtrate was applied directly to a 0.2-µm pore size of cellulose membrane filter (Advantec, Dublin, CA, USA). Following filtration, each Advantec membrane filter was stored at 4 °C until DNA isolation. eDNA isolation from seawater samples was performed on each of the filtered membranes using PowerWater^®^ DNA Isolation Kit (MOBIO, Qiagen, Carlsbad, CA, USA) and FastDNA^®^ SPIN Kit (MPbio, Santa Ana, CA, USA) following the manufacturer’s recommendations. eDNA from sediment samples was isolated using FastDNA^®^ SPIN Kit (MPbio). Approximately 50–100 ng of DNA was obtained from each sample. DNA quality was assessed by a 2100 Bioanalyzer (Agilent, Santa Clara, CA, USA) using an Agilent RNA 6000 Pico Kit (Agilent, Santa Clara, CA, USA). All the samples from the reservoirs were prepared using the 16S library preparation protocol and the Nextera XT DNA index kit (Illumina, San Diego, CA, USA) to target the V3–V4 variable region of the 16S rRNA gene for screening the bacterial biodiversity. A region of approximately 469 bp encompassing the V3–V4 hypervariable region was targeted for sequencing to maximize the effective length of the MiSeq’s 300-bp paired-end sequencing reads (see below). For the bacterial PCR amplification, the forward primer (5′-TCGTCGGCAGCGTCAGATGTGTATAAGAGACAG-CCTACGGGNGGCWGCAG-3′; ‘TCGTCGGCAGCGTCAGATGTGTATAAGAGACAG’ indicates the overhang adaptor sequences; the underlined sequence indicates the target region primer) and the reverse primer (5′-GTCTCGTGGGCTCGGAGATGTGTATAAGAGACAG-GACTACHVGGGTATCTAATCC-3′; ‘GT CTCGTGGGCTCGGAGATGTGTATAAGAGACAG’ indicates the overhang adaptor sequences; the underlined sequence indicates the target region primer) were used [29]. Library quantification was performed by real-time PCR using a CFX96 real-time system (BioRad, Hercules, CA, USA).

### 2.2. Sequencing and Prediction of 16S rRNA Data

The V3–V4 variable region of 16S rRNA was targeted and amplified. The amplicons were sequenced into 300-bp paired-end reads according to the Illumina MiSeq (San Diego, CA, USA) sequencing protocol. Raw data were trimmed using Trimmomatic v0.35 [30], and subsequently the quality control of raw data was included to filter out reads of quality scores <30 using FastQC v0.11.4 [31]. Each set of paired-end reads, which passed the quality check, was merged using FLASH v1.2.11 [32] to employ longer sequences.

### 2.3. Metagenome Predictions from 16S rRNA Data

Merged sequence data were analyzed in Quantitative Insights into Microbial Ecology (QIIME) 1.9.1 [33], an open-source bioinformatics pipeline for metagenome analysis. To perform each metagenome analysis for our experimental design, metadata and fasta files for each of the experiments were produced for applying in further analyses, along with the QIIME labeling step (add_qiime_labels.py). Next, operational taxonomic units (OTUs) were assigned with a similarity of 97% against Greengenes 13_8 [34] using uclust [35] through open-reference OTU assignment step including taxonomy assignment, sequence alignment, and tree building (pick_open_reference_otus.py). Using a summary of OTU-assignment step into biom-format (biom summarize), alpha and beta diversities that were calculated based on Unifrac distance matrix and principal coordinate analysis (PCA) were generated using the core diversity test step (core_diversity_analyses.py).

### 2.4. 16S rRNA Sequence Assembly and Statistical Analysis

Processing of raw data was performed by removing the overhang adaptor sequences that can be presented at the terminal of each read with Trimmomatic v0.33 using MiSeq NGS platform that targeted variable regions from V3 to V4 of the 16S rRNA gene. Reads having a quality score of <30 were excluded using FastQC v0.11.3, and trimmed sequence data were obtained (Table 1). Paired-end reads per sample were collapsed to a single read by combining each of the pair reads using FLASH v1.2.11. Metagenome analysis using QIIME is an open-source comprehensive program package that is used to analyze microorganism NGS data. Further, only the read that had the quality score higher than the specific standard was selected through quality filtering per merged sample, and analysis was performed for the samples by grouping them into surface layer (water), middle layer, deep layer, and sediment according to specific objectives of the analyses. However, the samples from surface and middle layers were combined before the formal analyses given the similar levels of diversity and species composition detected. OTUs corresponding to each read were searched using BLAST search (>97% identical, E-value < 10^−7^) based on the Greengenes 16S rRNA database, and each OTU number was normalized and provided through clustering according to each taxonomical standard (Table 1).

We statistically analyzed the number of OTUs using a Mann-Whitney *U* test to examine whether there was a significant difference in taxonomic richness between seawater (*n* = 12) and sediment (*n* = 3) microbial communities. An independent *t*-test was performed to test if there was a difference in the number of OTUs between the control (*n* = 6) and sand mining (*n* = 6) sites only for the seawater community. Another independent *t*-test was conducted to determine whether there was a difference in the number of OTUs between the spring (*n* = 6) and autumn (*n* = 6) samples. These analyses could not be done for the sediment community, owing to insufficient sample sizes.

## 3. Results

### 3.1. Differences in Taxonomic Richness between Sand Mining vs. Control Microbial Communities

**Figure 3:**
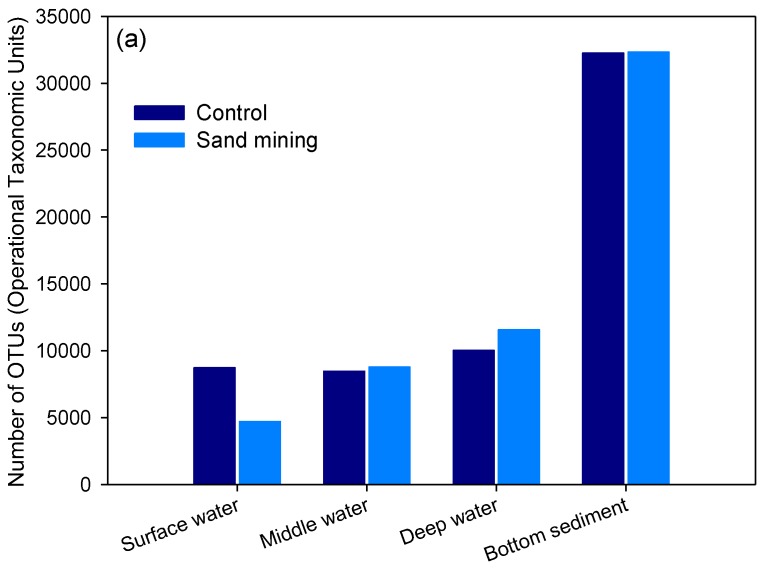

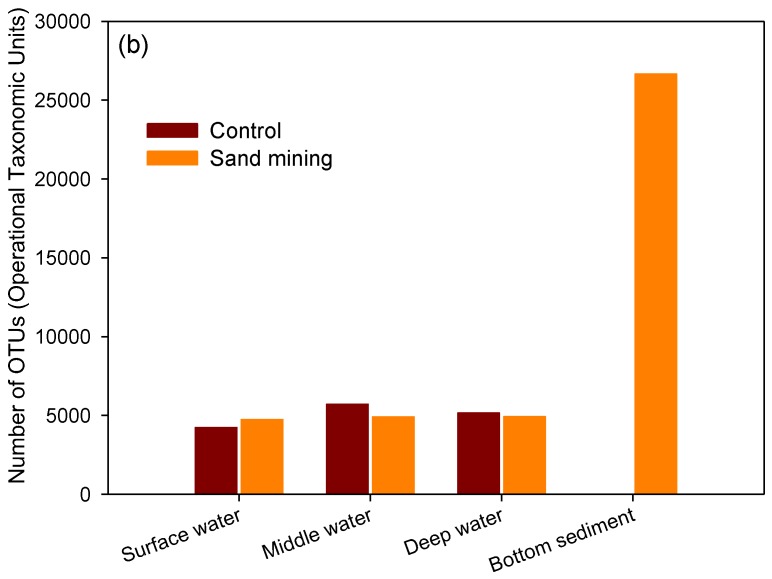
Differences in taxonomic richness in the seawater (surface, middle, deep layers) and sediment communities (as calculated by the number of Operational Taxonomic Units or OTUs) between sand mining and control sites during each of the sampling periods, April (**a**) and October (**b**) 2015.

The OTU number and the diversity of microorganisms found in the sediment community from the sea floor were significantly higher than those of the microorganisms found in the seawater community, irrespective of the sampling date and water depth (Mann-Whitney *U* = 0.00, *p* = 0.004) (Figure 3). The average number of OTUs was approximately four times greater for the sediment community (28,908) than for the seawater community (6828). Moreover, the number of OTUs in the seawater community was significantly higher for the spring samples than for the autumn samples (independent *t*-test, *t* = 3.955, *df* = 10, *p* = 0.003) (Figure 3).

The number of OTUs in the control site, however, did not significantly differ from that in the sand mining site (independent *t*-test, *t* = 0.294, *df* = 10, *p* > 0.05), despite noticeable differences observed in the composition and diversity between the two sites (see below). In the case of surface layer water from site 21 representative of the “sand mining” area, the average number observed per sample was the lowest at 4710 during April 2015, which was probably due to reduced microbial diversity caused by the anthropogenic disturbance to the surface water ecosystem by the increasing turbidity and toxicity (see below).

### 3.2. Differences in Composition and Diversity of Microbial Seawater Community between Sand Mining vs. Control Sites

**Figure 4:**
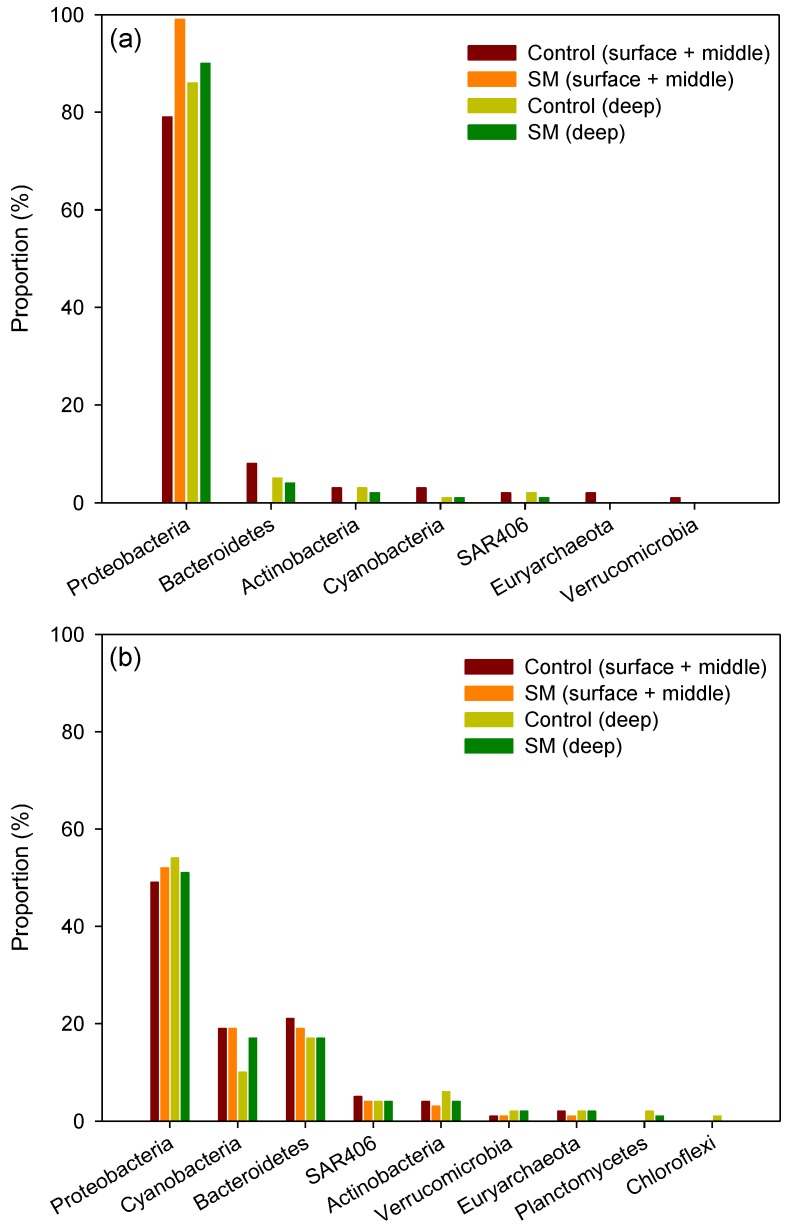
The phylum level taxonomic classification and comparison of bacterial reads of 16S rRNA from seawater samples (surface, middle, deep layers) between sand mining and control sites during the sampling periods of April (**a**) and October (**b**) 2015. SM: sand mining.

For the April 2015 samples, our results of microbial community in the samples from the surface seawater layer plus the middle layer revealed that site 12 (control) comprised microorganisms in the order of the phyla Proteobacteria (79%), Bacteroidetes (8%), Actinobacteria (3%), and Cyanobacteria (3%), while a simple composition of Proteobacteria (99%) was detected in site 21 (sand mining) (Figure 4a). In addition, samples from deep seawater layer in the control site consisted of microorganisms in the order of Proteobacteria (86%), Bacteroidetes (5%), and Actinobacteria (3%), whereas samples from the sand mining site consisted of microorganisms in the order of Proteobacteria (90%) and Bacteroidetes (4%) (Figure 4a).

**Figure 5:**
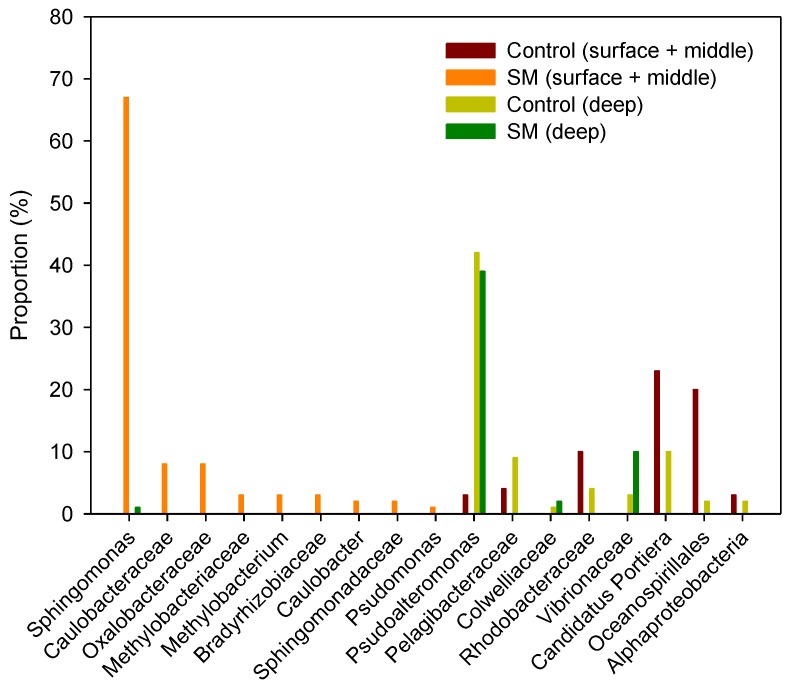
The subphylum level (family; genus) taxonomic classification and comparison of bacterial reads of 16S rRNA from seawater samples (surface, middle, deep layers) between sand mining and control sites during the sampling period of April 2015. SM: sand mining.

The community composition of the upper seawater layer ecosystems between the sand mining and control sites in spring turned out to be apparently divergent, although the microorganisms identified belonged to the same phylum Proteobacteria. The subphylum-level microbial community analysis for the samples from the surface seawater layer plus the middle layer showed that within the order Oceanospirillales, *Candidatus* Portiera and others comprised 43%, followed by Rhodobacteraceae at 10% in the control site, while samples from the sand mining site consisted of microorganisms in the order of Sphingomonadaceae (genus *Sphingomonas*; 67%), Oxalobacteraceae (8%), Caulobacteraceae (8%), Bradyrhizobiaceae (3%), and Methylobacteriaceae (3%) (Figure 5).

These results suggest that either *Sphingomonas* as a result of physical stress within sand mining areas was predominant as a response to degrading toxic compounds yielded by sand mining activities or Oxalobacteraceae and Caulobacteraceae were present in large quantities probably due to being engaged in nitrogen fixation ([17]; see below).

The results of the deep (seawater) layer microbial communities in April also showed that microorganisms were present in the order of *Pseudoalteromonas* (42%), *Candidatas* Portiere (10%), and Pelagibacteraceae (9%) in the control site, while those in the sand mining site were found in the order of Pseudoalteromonas (39%), Vibrionaceae (10%), and *Candidatas* Portiere (9%) (Figure 5). Vibrionaceae, which never appeared in the control site, was also observed, but its population was not as high as in the sample from the upper seawater community (e.g., surface layer plus middle layer), probably due to the relatively negligible effects of sand mining on the deeper water community.

The apparent differences in the structure of microbial communities between sand mining and control environments in April were rather uncertain in October (Figure 4b). Microbial community analysis at the phylum level from the surface and middle layer samples collected in October showed that microorganisms were present in the order of Proteobacteria (49%), Bacteroidetes (21%), and Cyanobacteria (19%) in the control site, whereas the microbial taxa composition in the sand mining site was in the order of Proteobacteria (52%), Bacteroidetes (19%), and Cyanobacteria (19%) (Figure 4b). For the deep seawater community, microorganisms were present in the order of Proteobacteria (54%), Bacteroidetes (17%), and Cyanobacteria (10%) in the control site, while those in the sand mining site were found in the order of Proteobacteria (51%), Bacteroidetes (17%), and Cyanobacteria (17%).

Again, there were no obvious differences in the compositions of microorganisms between the samples collected in October at the subphylum level (family, genus) (Figure S1, Electronic Supplementary Material). When compared with the samples collected in April, the number of Proteobacteria was significantly reduced, suggesting that seasonal differences existed (Figure S1). Furthermore, microorganism community analysis at the subphylum level for the surface plus middle layer samples collected in October showed that microorganisms were found in the order of Pelagibacteraceae (16%), Synechococcaceae (16%), Flavobacteriaceae (9%), Alphaproteobacteria (9%), and Rhodobacteraceae (8%) in the control site, while those in the sand mining site were found in the order of Synechococcaceae (17%), Pelagibacteraceae (15%), and Flavobacteriaceae (9%). Deep layer samples collected in October contained microorganisms in the order of Pelagibacteraceae (18%), Synechococcaceae (9%), Flavobacteriaceae (7%), and Alphaproteobacteria (7%) in the control site, while microorganisms in the sand mining site were found in the order of Pelagibacteraceae (17%), Synechococcaceae (15%), Flavobacteriaceae (8%), and Alphaproteobacteria (8%), indicating the abrupt increases of Synechococcaceae in comparison with those in the control site (Figure S1).

### 3.3. Differences in Composition and Diversity of Microbial Sediment Community between Sand Mining vs. Control Sites

**Figure 6:**
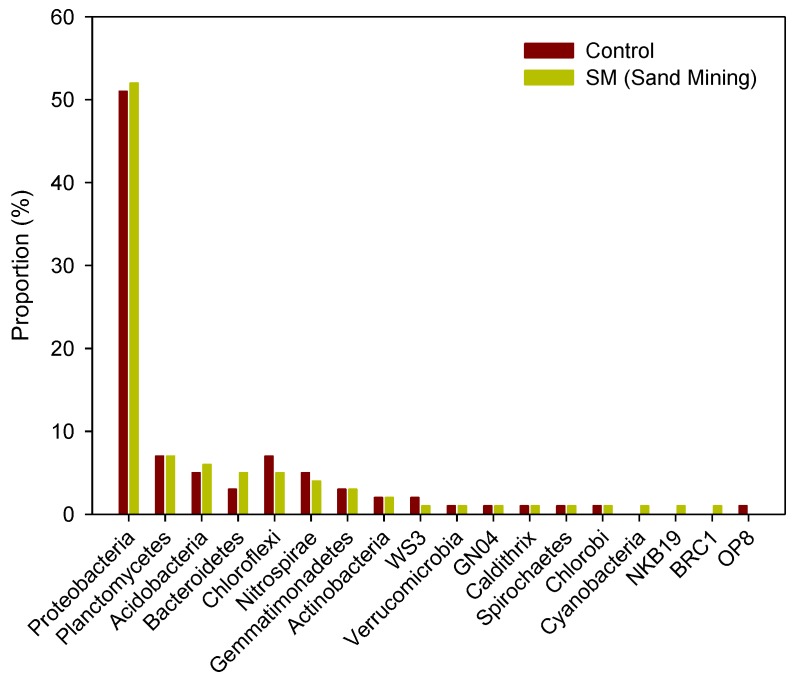
The phylum level taxonomic classification and comparison of bacterial reads of 16S rRNA from bottom sediment samples between sand mining (SM) and control sites during the sampling period of April 2015.

For the microbial sediment community, phylum-level analysis of the April samples showed that microorganisms were found in the order of Proteobacteria (50%), Planctomycetes (7%), Chloroflexi (7%), and Acidobacteria (6%) in the control site, while microorganisms in the sand mining site were found in the order of Proteobacteria (51%), Planctomycetes (7%), Acidobacteria (6%), and Chloroflexi (5%), indicative of similar community composition between the sites (Figure 6). Accordingly, composition of the microbial community at the subphylum level from the sediment samples collected in April was also similar between the sites as seen in the phylum-level community analysis (Figure S2).

Besides, microorganism composition from the samples collected in three replications only from the site SM (sand mining) in October was in the order of Proteobacteria (50.6%), Planctomycetes (9%), and Acidobacteria (5.6%). Results of genus-level microbial community analysis for the sediment samples collected in October showed that microorganisms were found in the order of Piscirickettsiaceae (6%), *Desulfococcus* (4.3%), and Alphaproteobacteria (3%), showing no apparent differences in the dominant taxonomic groups by season or site. These results can be attributed to less significant effects of seasonal or sand mining on the sediment microbial community as compared with that in the seawater, but the overall OTU numbers were drastically reduced in terms of taxonomic diversity compared with those collected in April (Figure 3).

Furthermore, unique microorganisms were categorized by the depth of seawater and sediment occurring only in the samples from the sand mining sites (Table 2 and Table 3). The large number of microbial taxonomic groups showed that the dominance of *Micrococcus* is probably due to physical stress within the sand mining sites, and that *Sphingomonas* might be involved in toxic compound degradation (Figure 5; [36]), and Oxalobacteraceae and Caulobacteraceae are engaged in nitrogen fixation. Moreover, several groups of microorganisms are involved in hydrocarbon degradation (*Marinobacter*), oil degradation (*Alcanivorax*), aromatic compound degradation (*Novosphingobium*), sulfate-methane zone existence (*Desulfofaba)*, or potential pathogens (*Parabacteroides, Coxiella*).

## 4. Discussion

Microbial communities are an essential component of coastal ecosystems as they play an indispensable role in ecosystem functioning and providing services such as biogeochemical cycles, degradation of organic materials, energy production, food web dynamics, and climate change regulation [6]. In particular, coastal ecosystems harbor large numbers of microorganisms, which may be an order of magnitude higher with higher productivity and higher load of organic matter and nutrients, compared with any other marine systems [6]. Coastal microbial communities are highly dynamic over space and time in response to environmental heterogeneities such as day length, temperature, water depth, oceanic currents, and inorganic nutrients [13,14]. During the last decade, a number of studies on natural microbial communities in marine environments have been performed using an NGS-based metagenomics approach to explore the diversity, community structure or composition, and its seasonal variation from various oceanic regions worldwide (e.g., [13,23,24,28,37,38]), yet, relatively little attention has been paid to the impacts of anthropogenic disturbances such as “sand mining” activity on the marine ecosystem using genomic DNA analysis from a whole microbial community [9,10,18,19]. A recent study with a metagenomics approach revealed that shifts in microbial composition within Sydney Harbor, Australia, which is one of the most anthropogenically highly impacted urban estuaries, were strongly linked to an enrichment of total microbial metabolic pathways including phosphorus and nitrogen metabolism, sulfate reduction, virulence, and the degradation of hydrocarbons [9]. Sand dredging is suggested to have a remarkable influence on the entire marine community in the North Sea comprised of phyto- and zooplanktons, suspended, benthic filter feeders and pelagic fish [18]. There are few studies that have examined the natural microbial communities in the Korean marine ecosystems [14], although a few results have so far been documented by applying metagenomics to screen or characterize functionally significant enzymes or biochemicals from the mud flat ecosystem [39,40]. Our study is the first, to our knowledge, to explore the structure and diversity of microbial communities in the Korean seawaters, with a focus on the impacts of anthropogenic disturbance, i.e., sand mining activity, on the coastal ecosystem. Although the number of our study sites analyzed is rather small (one each for the sand mining and control environments), our results show that sand mining regions had an apparent loss in microbial taxonomic diversity and also differed in bacterial composition, especially in surface water communities in spring season. Sand mining activity is now being performed year-round on a daily basis under the legislative control by the Korean government since 2004 depending on oceanographic conditions. Therefore, it would be conceivable that the observed differences in the microbial diversity and composition between the sand mining and control environments might be attributed to the overflowing of pumped bottom sediment driven by dredging, resulting in increasing turbidity and toxicity [17]. However, future studies with more extensive sampling (e.g., seawater sampling of moving water flow via the ocean currents rather than that of a fixed spot) are certainly required to better understand the “in situ” effects of sand mining disturbances on the target ecosystem [13]. The results of the analysis of the structure and diversity of microbial communities in the seawater and sediment environments reported here will represent the basis for future efforts on understanding the ecological or environmental factors that play a role in shaping microbial communities in the Korean waters.

The bacterial taxonomic diversity as estimated by the number of OTUs is far greater in the sediment community than in the seawater community, regardless of the sampling period and location (Figure 3). A previous study on the composition of microbial assemblages in the Changjiang estuary and coastal regions of the East China Sea found that bacterial diversity in the sediment samples was much greater than that in the seawater samples and also Proteobacteria (72.9%) was the most dominant phylum in the sediment [37]. Moreover, sediment from the Antarctic continental shelf consisted of Proteobacteria as the most predominant bacterial lineage comprising >50% of the microbial biomass analyzed [41]. Similarly, our results also indicate that Proteobacteria is the most abundant phylum and comprises approximately 50% of the sediment bacterial assemblages in both the sand mining and control sites. However, this trend was not observed in deep-sea offshore oceans; taxonomic richness was found to be generally highest in the surface seawater and lowest in the seafloor sediment for deep-sea environments [38].

The reduced microbial diversity and the changes in the composition of the bacterial assemblages in the perturbed area can be attributed to the complicated impacts of the possible pollutants including suspended particles, nutrients, organic pollutants and heavy metals, entailed by the sediment load and dredging, i.e., sand mining activity (Figure 1) [17]. Previous studies suggest that heavy metals and organic compounds contamination through sand mining or dredging activities originate from polluted bottom sediments in the North Sea [18,19]. The data obtained from the sample of both the surface and middle seawaters from the sand mining site collected in April strongly support such a trend. There are prominent differences in the microbial diversity at the phylum and subphylum (family and genus) levels. For example, surface and middle seawater microbial communities at the sand mining site during April comprised exclusively Proteobacteria (99%), while those at the control site consisted of only 79% of Proteobacteria (Figure 4a). Furthermore, the phyla Bacteroidetes (8%), Actinobacteria (3%), Cyanobacteria (3%), Euryarchaeota (2%), and Verrucomicrobia (1%) were found in the surface and middle seawater communities at the control site, but none were found in the sand mining site. These results of predominance of Proteobacteria in the seawater regardless of location are consistent with previous findings that this phylum comprised 63%–95% of the bacterial communities year-round in Gosung Bay on the southern coast of Korea [14]. Plausible ecological impacts involved with the sediment load/dredging include increasing turbidity, the resulting decrease in light penetration and nutrient enrichment, and toxicity [17]. The nonappearance of phototrophic microorganisms, *Cyanobacteria*, in the upper seawater communities in the sand mining sites suggests that suspended particles by dredging certainly blocked light absorption into the upper water layer, which results in the lack of phototrophic bacteria there. The differences in water reflectance observed between the sand mining and control sites based on satellite data from Geostationary Ocean Color Imager (GOCI) can support this hypothesis (N. Won, unpublished data). However, the microbial diversity and composition in the samples collected in October did not greatly differ when compared with those in April. These results might be due to seasonal changes in hydrography, such as movement of seawater flow and other environmental factors including day length [13], water temperature [12], salinity, and inorganic nutrients such as phosphate and nitrate concentrations [14]. The lack of differences in the composition and diversity of microbial assemblages between the sand mining vs. control sites in October is possibly due to the mixed overwhelming effects of typhoons with strong winds and storms on the study ecosystem [16,42].

In the case of the sediment microbial community, the composition and diversity of dominant taxonomic groups did not greatly change as those in the seawater community by season and location. These findings might suggest that the sediment bacterial community from the sea floor is perhaps more stable over space and time [38] or even more resilient following devastating disturbances such as sand mining activities. However, this hypothesis needs to be validated in further studies.

Our study allows for identifying the microbial taxonomic groups exclusively occurring in the sand mining sites (Table 2 and Table 3). Interestingly, several groups of microorganisms solely detected in the seawater communities from the disturbed areas are known to be involved in the degradation of toxic chemicals including hydrocarbons (*Marinobacter*) [43], oil (*Alcanivorax*) [44], aromatic compounds (*Novosphingobium*) [45], sulfate-methane reduction (*Desulfofaba*), or they possess pathogenic characteristics (*Parabacteroides, Coxiella*). Similarly, some bacterial groups occurring only at the bottom sediments from the sand mining site have chemoorganotrophic (*Geosporobacter*), hydrocarbonoclastic (*Oleispira*), or pathogenic (*Legionella*) features. These results are consistent with a previous report that sediment load and dredging can give rise to toxicity and biopollution in marine environments [17].

## 5. Conclusions

In this study, using a metagenomic DNA sequencing (16S rRNA gene) approach, we showed that the human disturbance and the sand mining activity, can result in a remarkable loss of diversity and changes in the composition of microbial surface seawater assemblages in the South Sea of Korea. We also found that the microbial taxonomic groups present only in the perturbed environments are involved in pollutant-associated biological processes. The findings reported here can be directly applied to the evaluation and monitoring of the impacts of human-mediated environmental pollution on the distribution and diversity of microbial groups in the marine ecosystems in the future.

## Figures and Tables

**Table 1:** Information of raw sequences of 16S ribosomal RNA (rRNA) gene from the sea water (surface, middle and deep layers) and bottom sediment samples using Next Generation Sequencing (NGS)-MiSeq platform. St 21 and St SM (sand mining) represent “sand mining” sites and St 12 “control” site.

Site ID	Latitude; Longitude	Sample Type	Samples	Sampling Period	Read Counts	Total Base Pair Counts
Site12	34°15.05′ N; 128°23.68′ E	Control	Surface	April 2015 (Spring)	876,484	263,821,684
			Middle		904,794	272,342,994
			Deep		865,924	260,643,124
Site21	34°11.55′ N; 128°22.55′ E	Sand mining	Surface		887,080	267,011,080
			Middle		574,816	173,019,616
			Deep		511,724	154,028,924
Site12	34°15.05′ N; 128°23.68′ E	Control	Sediment		845,526	254,503,326
Site21	34°11.55′ N; 128°22.55′ E	Sand mining	Sediment		741,150	223,086,150
Site12	34°15.05′ N; 128°23.68′ E	Control	Surface	October 2015 (Autumn)	845,526	254,503,326
			Middle		1,260,066	379,279,866
			Deep		1,039,742	312,962,342
Site SM	34°11.17′ N; 128°24.50′ E	Sand mining	Surface		1,135,076	341,657,876
			Middle		1,052,748	316,877,148
			Deep		1,133,566	341,203,366
Site SM		Sand mining	Sediment		1,090,470	328,231,470
			Sediment		1,496,222	450,362,822
			Sediment		1,955,034	588,465,234

**Table 2:** Microbial taxonomic groups that were only detected in the seawater samples from the sand mining sites.

Sample	Class	Family	Genus
Surface water	Cenarchaeales	Cenarchaeaceae	-
	Flavobacteriales	Flavobacteriaceae	*Mesonia*
	Ignavibacteriales	Ignavibacteriaceae	*-*
	Kordiimonadales	Kordiimonadaceae	*-*
	Burkholderiales	Alcaligenaceae	*-*
	Alteromonadales	Alteromonadaceae	***Marinobacter***
	Alteromonadales	Idiomarinaceae	*Pseudidiomarina*
	Oceanospirillales	Alcanivoracaceae	***Alcanivorax***
	Oceanospirillales	Halomonadaceae	*Cobetia*
	Marinicellales	Marinicellaceae	*Marinicella*
Middle water	Bacteroidia	Bacteroidaceae	*Bacteroides*
	Bacteroidia	Porphyromonadaceae	***Parabacteroides***
	Flavobacteriia	Flavobacteriaceae	*Cellulophaga*
	Flavobacteriia	Weeksellaceae	*-*
	Clostridia	Lachnospiraceae	*Lachnospira*
	Clostridia	Veillonellaceae	*Succiniclasticum*
	Clostridia	Acidaminobacteraceae	*Fusibacter*
	Fusobacteriia	Fusobacteriaceae	*Propionigenium*
	Alphaproteobacteria	Erythrobacteraceae	*-*
	Alphaproteobacteria	Sphingomonadaceae	***Novosphingobium***
	Deltaproteobacteria	Desulfarculaceae	*-*
	Deltaproteobacteria	Desulfobacteraceae	***Desulfofaba***
Deep water	Actinobacteria	Micrococcaceae	*Micrococcus*
	Cytophagia	Amoebophilaceae	*Ucs1325*
	Chlamydiia	Simkaniaceae	*-*
	Ignavibacteria	lheB3-7	*-*
	Synechococcophycideae	Synechococcaceae	*-*
	Bacilli	Streptococcaceae	*Streptococcus*
	Alphaproteobacteria	Kiloniellaceae	*Thalassospira*
	Alphaproteobacteria	Rhodospirillaceae	*-*
	Alphaproteobacteria	Erythrobacteraceae	*Erythrobacter*
	Deltaproteobacteria	Desulfarculaceae	*-*
	Deltaproteobacteria	Desulfobacteraceae	*Desulfosarcina*
	Deltaproteobacteria	Syntrophaceae	*Desulfobacca*
	Gammaproteobacteria	Coxiellaceae	***Coxiella***
	Gammaproteobacteria	Halomonadaceae	*Haererehalobacter*
	Gammaproteobacteria	Oceanospirillaceae	*Oleibacter*
	PRR-12	KSB4	-

The genus in bold is known to be involved in toxic chemical degradation or pathogenic pollution (see Results and Discussion).

**Table 3:** Microbial taxonomic groups that were only detected in the bottom sediment samples from the sand mining sites.

Sample	Class	Family	Genus
Bottom sediments	Actinobacteria	Bifidobacteriaceae	*Bifidobacterium*
	Fimbriimonadia	Fimbriimonadaceae	*-*
	Bacteroidia	Bacteroidaceae	*Bacteroides*
	Flavobacteriia	Cryomorphaceae	*-*
	Flavobacteriia	Cryomorphaceae	*Cryomorpha*
	Flavobacteriia	Cryomorphaceae	*Owenweeksia*
	Rhodothermi	Balneolaceae	*Balneola*
	Synechococcophycideae	Synechococcaceae	*Prochlorococcus*
	Bacilli	Planococcaceae	*Sporosarcina*
	Bacilli	Thermoactinomycetaceae	*-*
	Clostridia	Clostridiaceae	*Alkaliphilus*
	Clostridia	Clostridiaceae	***Geosporobacter***
	Clostridia	Lachnospiraceae	*-*
	Clostridia	Lachnospiraceae	*Coprococcus*
	Clostridia	Lachnospiraceae	*Lachnospira*
	Clostridia	Peptostreptococcaceae	*-*
	Clostridia	Ruminococcaceae	*Faecalibacterium*
	Clostridia	Ruminococcaceae	*Ruminococcus*
	Clostridia	Veillonellaceae	*Dialister*
	Clostridia	Veillonellaceae	*Megamonas*
	Clostridia	Acidaminobacteraceae	*-*
	Fusobacteriia	Fusobacteriaceae	*u114*
	Alphaproteobacteria	Methylocystaceae	*Pleomorphomonas*
	Alphaproteobacteria	Rhodobiaceae	*Afifella*
	Alphaproteobacteria	Rhodobacteraceae	*Marivita*
	Alphaproteobacteria	Rhodobacteraceae	*Thalassobius*
	Alphaproteobacteria	Rhodospirillaceae	*Novispirillum*
	Alphaproteobacteria	Rhodospirillaceae	*Rhodospirillum*
	Alphaproteobacteria	AEGEAN_112	*-*
	Deltaproteobacteria	S25_1238	*-*
	Deltaproteobacteria	SAR324	*-*
	Gammaproteobacteria	Psychromonadaceae	*-*
	Gammaproteobacteria	Legionellaceae	***Legionella***
	Gammaproteobacteria	Legionellaceae	*Tatlockia*
	Gammaproteobacteria	Oceanospirillaceae	***Oleispira***
	AB16	A714017	*SargSea-WGS*
	Mollicutes	Mycoplasmataceae	*Candidatus Hepatoplasma*

The genus in bold is known to be involved in toxic chemical degradation or pathogenic pollution (see Results and Discussion).

## Supplementary Materials

The following are available online at www.mdpi.com/1660-4601/14/2/130/s1, Figure S1: The subphylum level (family; genus) taxonomic classification and comparison of bacterial reads of 16S rRNA from seawater samples (surface, middle, deep layers) between sand mining and control sites during the sampling period of October 2015, Figure S2: The subphylum level (family; genus) taxonomic classification and comparison of bacterial reads of 16S rRNA from sediment samples between sand mining and control sites during the sampling period of April 2015, Figure S3: Graphical representations of environmental parameters (temperature (°C), salinity (practical salinity unit, PSU) and density (Sigma t, kg/m3)) obtained during sampling periods of spring and autumn 2015 from the sand mining (SM) and control sites. Note that control site was not identical to the site (St. 12) where seawater and sediment samples were collected. The environmental data are given as follows: station 1 (SM) in spring (A), station 5 (control) in spring (B), station 1 (SM) in autumn (C) and station 6 (control) in autumn (D).
