# Supplementary material for: Exploring the Impacts of Anthropogenic Disturbance on Seawater and Sediment Microbial Communities in Korean Coastal Waters Using Metagenomics Analysis
Source: Int J Environ Res Public Health. 2017 Jan 27;14(2):130. doi: 10.3390/ijerph14020130 (PMC5334684; doi:10.3390/ijerph14020130)
Supplement: Supplementary file 1 [file ijerph-14-00130-s001.pdf]

Supplementary Materials: Exploring the Impacts of Anthropogenic Disturbance on Seawater and Sediment Microbial Communities in Korean Coastal Waters Using Metagenomics Analysis

Nam-Il Won, Ki-Hwan Kim, Ji Hyoun Kang, Sang Rul Park and Hyuk Je Lee

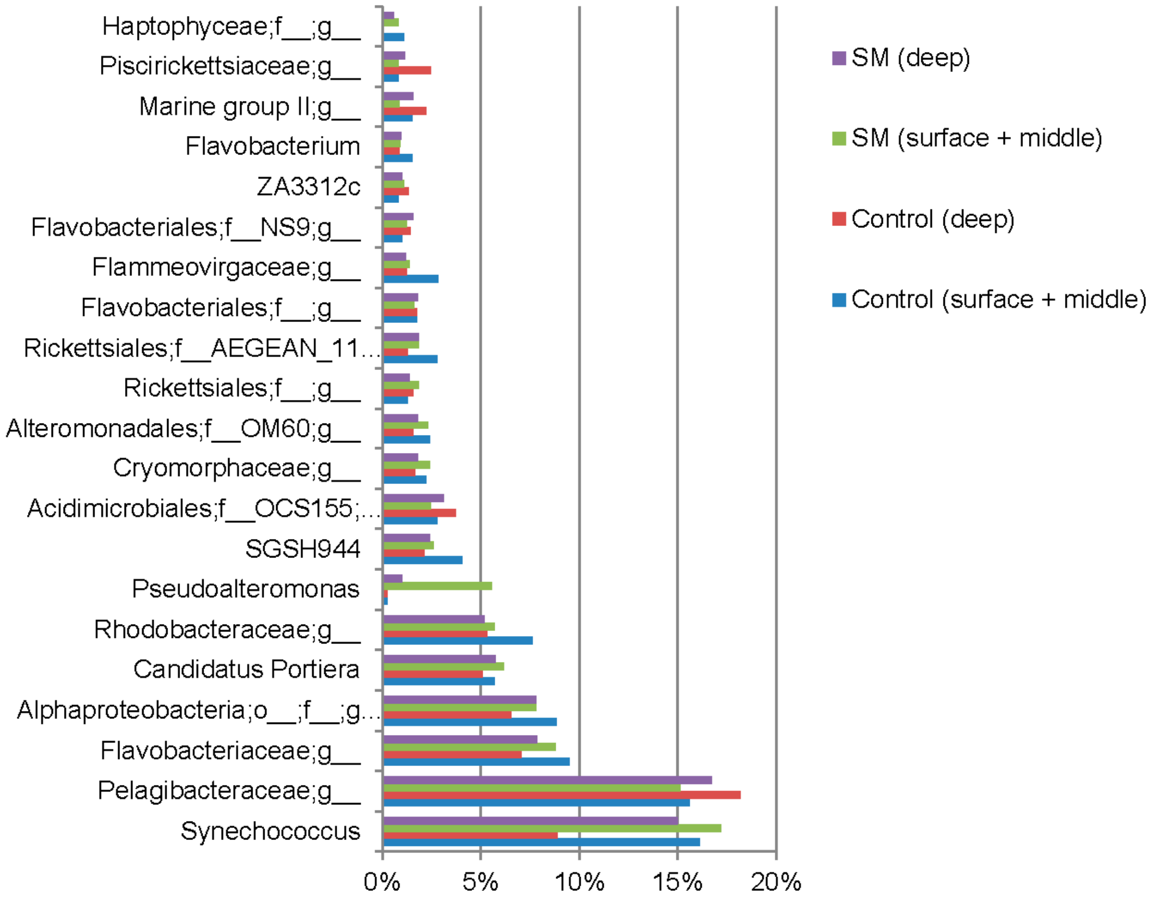

Figure S1. The subphylum level (family; genus) taxonomic classification and comparison of bacterial reads of *16S rRNA* from seawater samples (surface, middle, deep layers) between sand mining and control sites during the sampling period of October 2015. SM: sand mining.

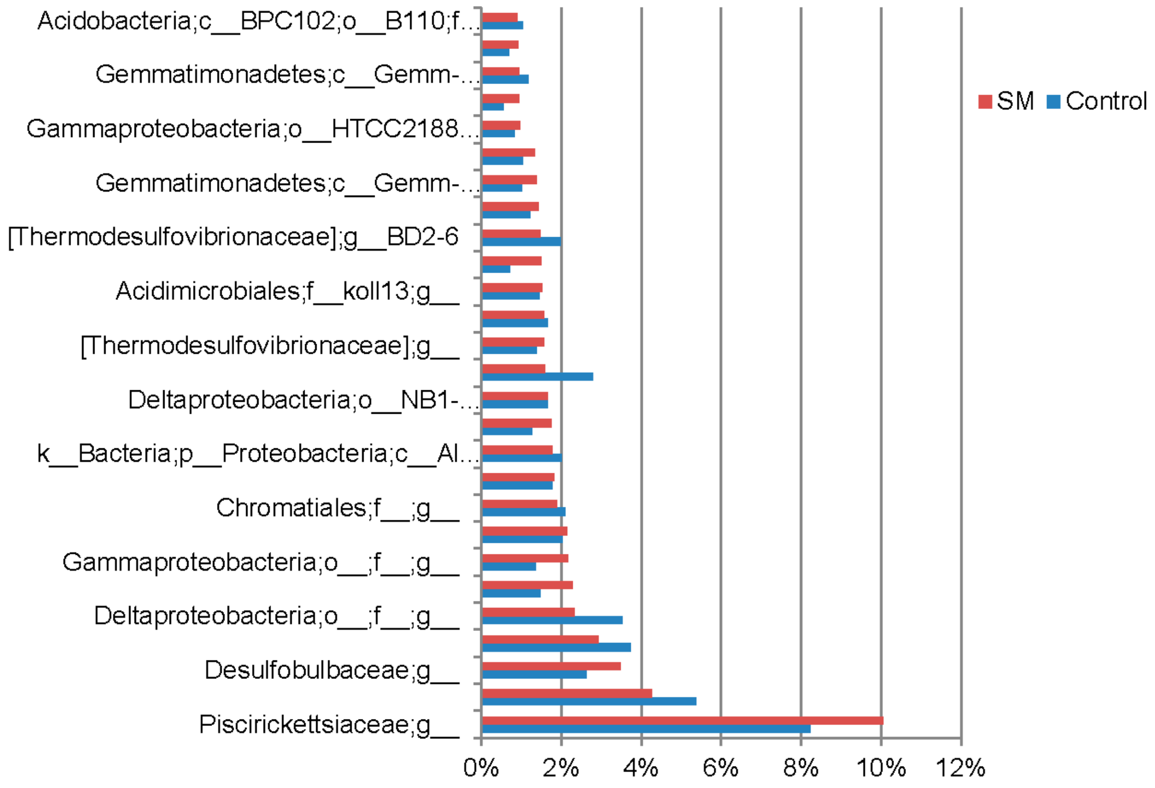

Figure S2. The subphylum level (family; genus) taxonomic classification and comparison of bacterial reads of *16S rRNA* from sediment samples between sand mining and control sites during the sampling period of April 2015. SM: sand mining.

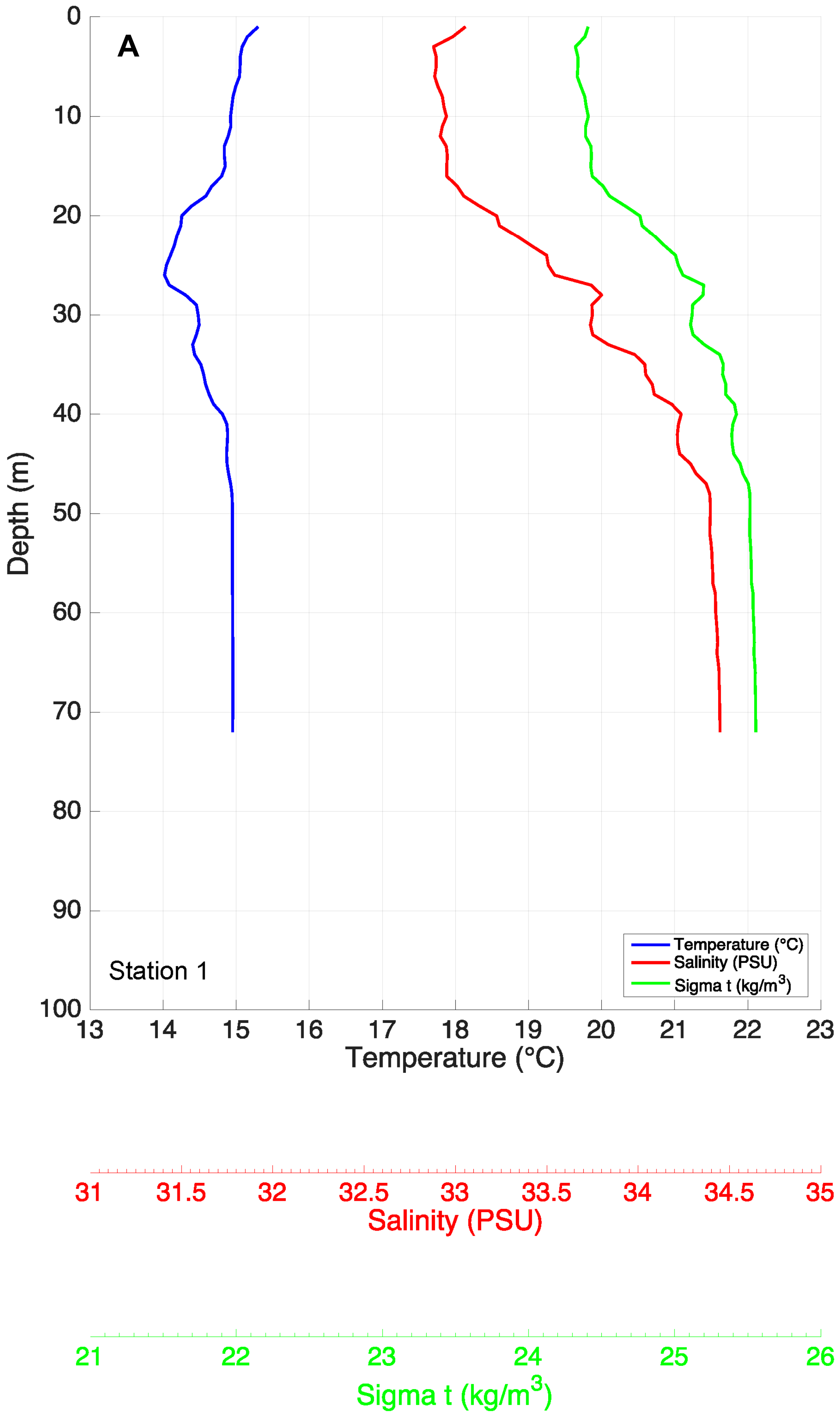

Figure S3. Cont.

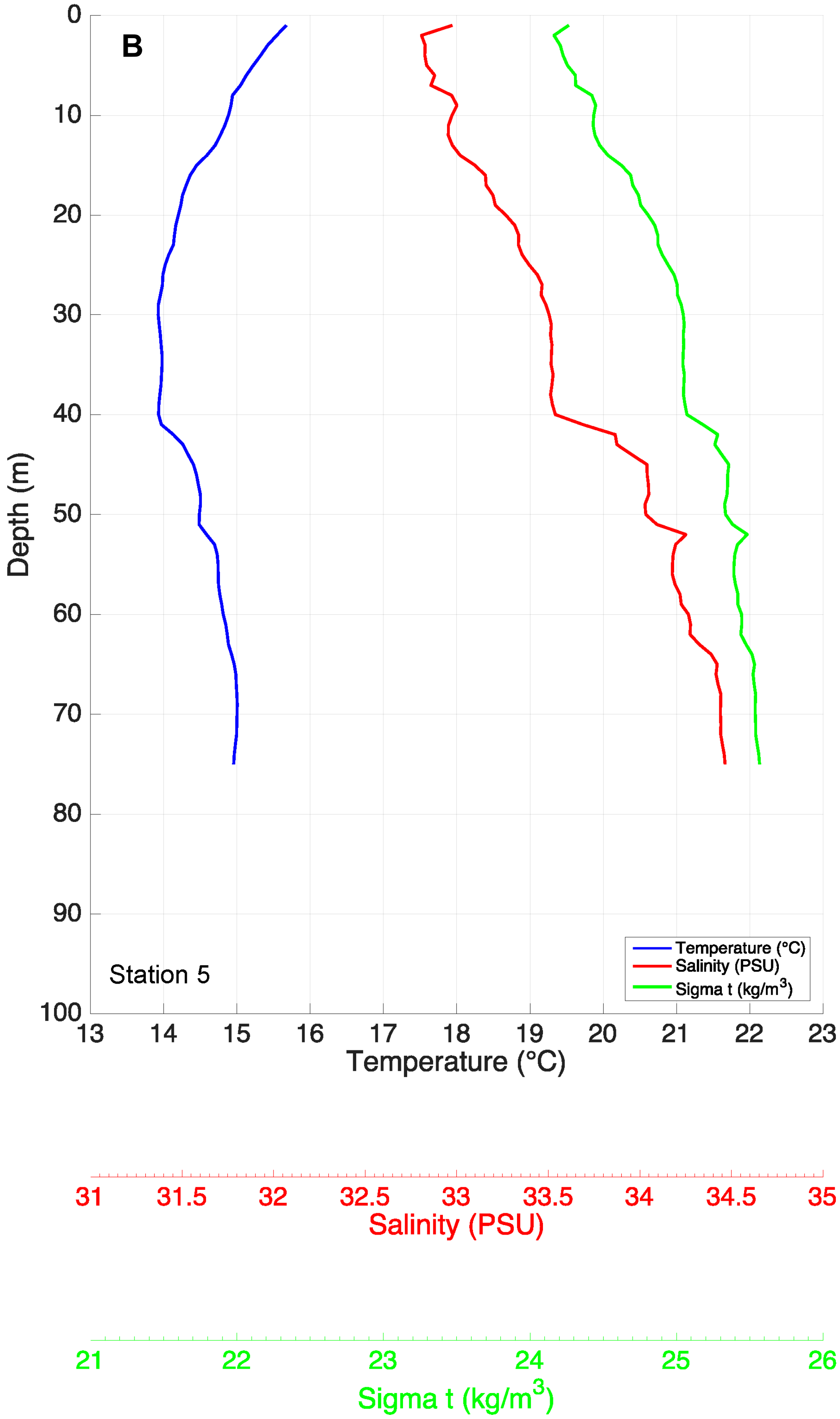

Figure S3. Cont.

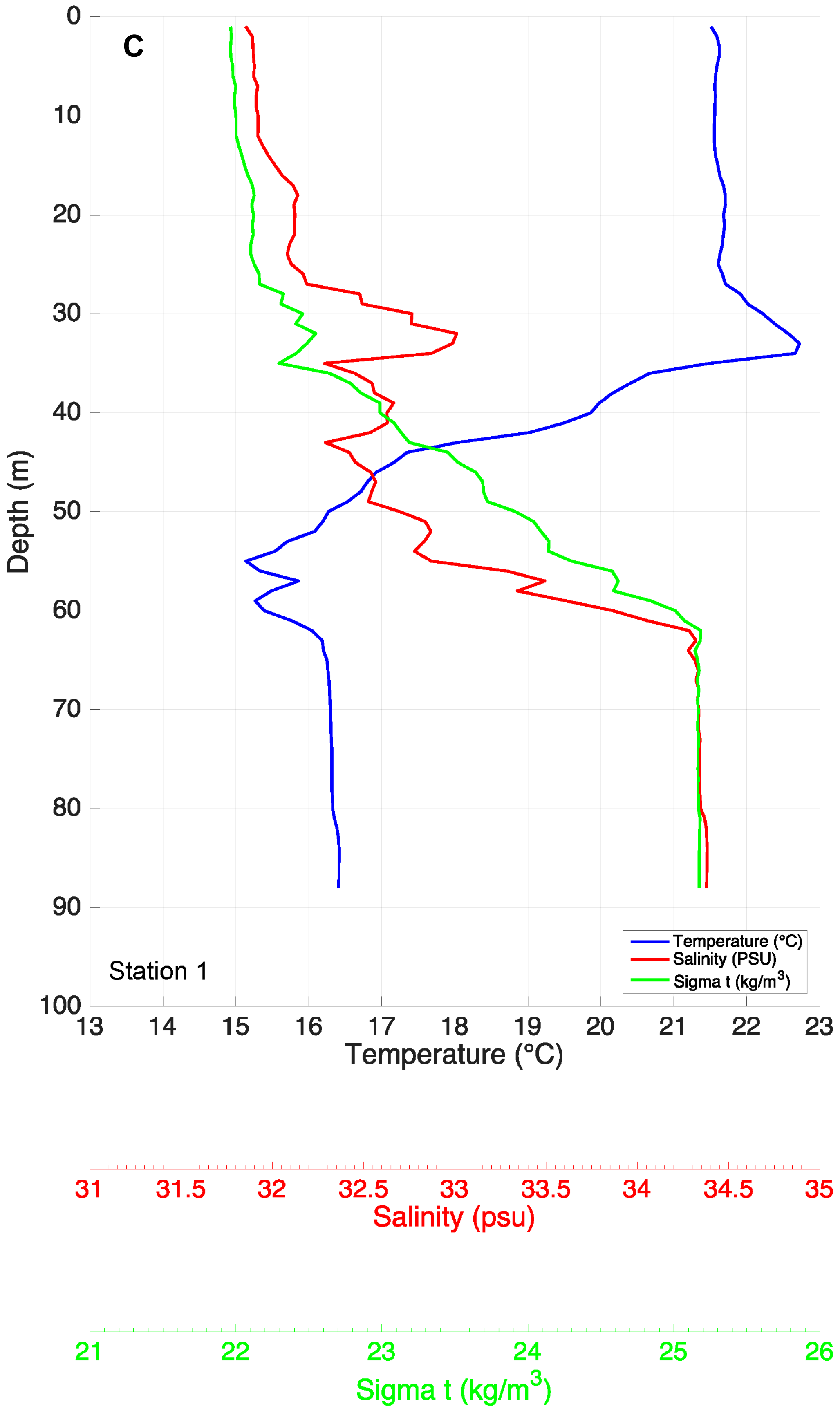

Figure S3. Cont.

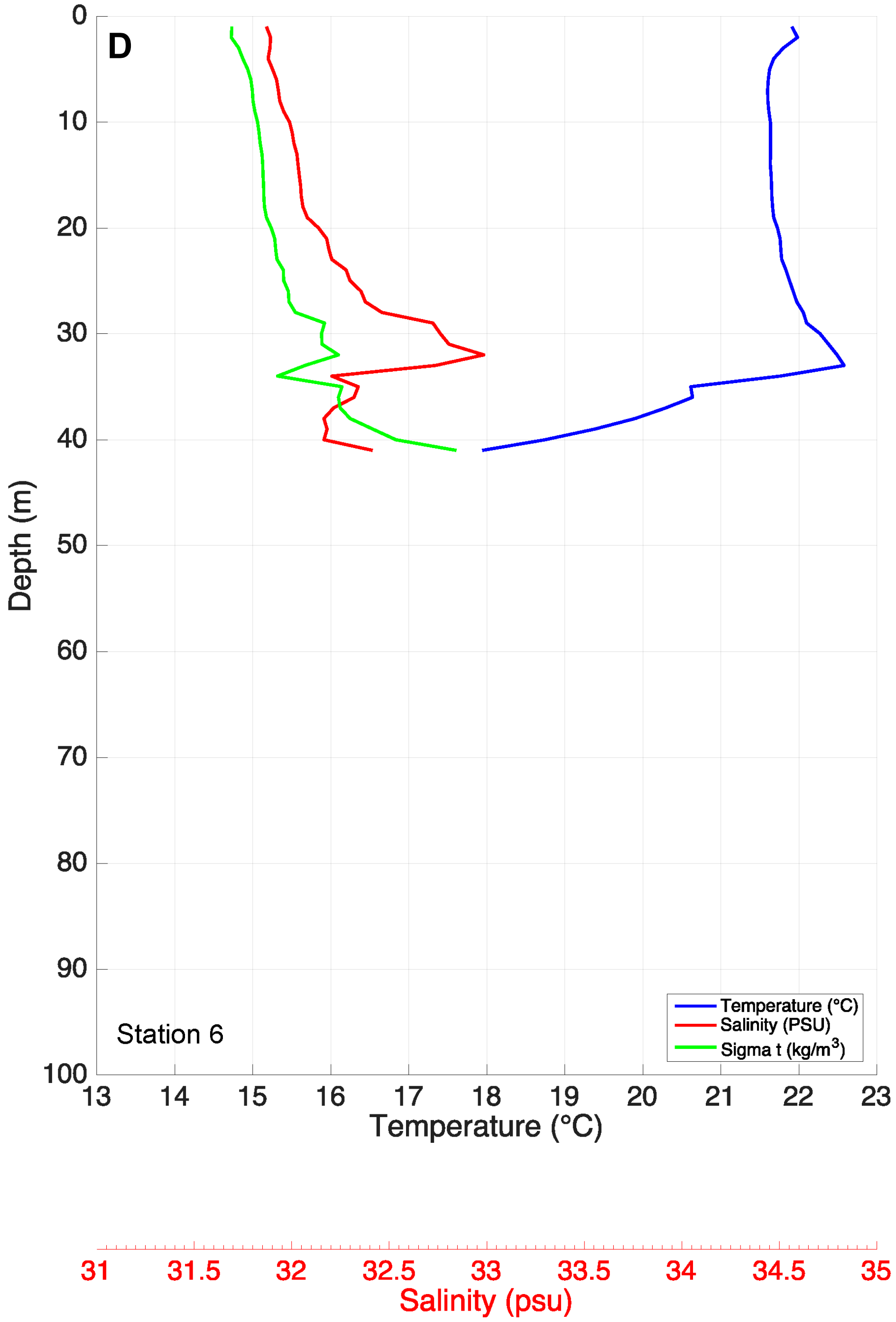

Figure S3. Graphical representations of environmental parameters (temperature (°C), salinity (practical salinity unit, PSU) and density (Sigma t, kg/m^3)) obtained during sampling periods of spring and autumn 2015 from the sand mining (SM) and control sites. Note that control site was not identical to the site (St. 12) where seawater and sediment samples were collected. The environmental data are given as follows: station 1 (SM) in spring (A), station 5 (control) in spring (B), station 1 (SM) in autumn (C) and station 6 (control) in autumn (D).

© 2017 by the authors; licensee MDPI, Basel, Switzerland. This article is an open access article distributed under the terms and conditions of the Creative Commons by Attribution (CC-BY) license (http://creativecommons.org/licenses/by/4.0/).
